# An ethnographic study evaluating emergency obstetric care education and training in a remote, fragile region of Southeast Asia: Study protocol

**DOI:** 10.1002/nop2.2034

**Published:** 2023-10-14

**Authors:** Rachel Campbell, George Kernohan, Lesley Dornan, Marlene Sinclair

**Affiliations:** ^1^ The Centre for Maternal, Fetal and Infant Research Institute of Nursing and Health Research, Ulster University Belfast UK

**Keywords:** fragile, health personnel, maternal health, obstetric emergencies, remote, Southeast Asia, training

## Abstract

**Aim:**

To evaluate emergency obstetric care education and training within a medical assistant training program, being delivered in a remote, fragile region of Southeast Asia. This will aid in the identification of potential areas of enhancement to improve the management of obstetric emergencies.

**Design:**

An ethnographic study, adopting a multi‐methods approach.

**Methods:**

Emergency obstetric care education and training will be assessed through documentary analysis and interviews (online or face‐to‐face) with educators and trainers (*N* ~ 6–7). Student experiences will be explored using in‐person focus groups, facilitated by an external trainer involved in delivering the program (*N* ~ 10–12). A reflective field diary will provide insight into the lived experience of postgraduate students (*N* ~ 4–5). Data will be collected between May 2022 and May 2023. The full data set will be triangulated and analysed using the READ approach; (1) ready your materials, (2) extract data, (3) analyse data and (4) distil your findings. Institutional ethical approval was obtained from a university in October 2021, and inter‐country regional access was gained following adherence to their local ethical requirements.

**Implications for the Profession and Patient Care:**

The findings from this study may help to inform the future design of the medical assistant training program. It is anticipated that the knowledge gained from this study will enhance the education and training of mid‐level health providers at local, national, and international levels. This work intends to contribute to addressing Sustainable Development Goal 3, Target 1 of reducing maternal mortality to 70:100,000 live births in low‐income countries.

**Reporting Method:**

This protocol adhered to the Standards for Reporting Qualitative Research (SRQR) checklist.

**Patient or Public Contribution:**

No formal PPI has been undertaken; however, stakeholders involved in delivering the education and training have been consulted.

## 1 INTRODUCTION

This protocol outlines an ethnographic study, which will be conducted as part of a wider project “Birth Across the Borders (BAB)” (UK Research and Innovation, 2022). The BAB project is working closely with local partners, with the intent to gain a deeper understanding of the specific challenges facing families living within four remote regions in Myanmar. It involves the co‐development of three educational programmes, specifically adapted for each region. The first package focuses on helping families and communities to recognize obstetric complications, and subsequently know where to go to access help. The second package aims to further train health workers to recognize the signs of obstetric complications and how to effectively manage these emergencies. The third package considers entrepreneurship, to help support local people and community leaders to set up their own business, enabling them to move out of poverty and access the healthcare they require. This three‐step integrated approach is framed on the Three Delays Model (Thaddeus & Maine, 1994) and aims to reduce preventable maternal and neonatal deaths by addressing the issues women face when trying to access safe childbirth in remote, fragile regions of Myanmar.

### 1.1 Contribution to wider project

This ethnographic study falls within work package two of the wider project and aims to address the third issue identified in the Three Delays Model (Thaddeus & Maine, 1994), which is a delay in receiving adequate health care. The World Health Organization (WHO) highlights the important role mid‐level health providers (MLHPs) can play in the scale‐up of the health workforce within remote regions (WHO, 2017). Although MLHPs are not medical doctors, their role is multifaceted and usually includes tasks carried out by doctors, nurses, and midwives. Various titles fall under the umbrella of MLHPs; however, for the purpose of this study, the training program being evaluated will be referred to as a medical assistant training program.

Building upon partnerships consolidated over 5 years of international networking and research, facilitated by the wider BAB project, this study will be conducted in partnership with an organization delivering a medical assistant training program in a remote, fragile region of Myanmar. This study aims to evaluate the current level of emergency obstetric care (EmOC) education and training being delivered as part of the medical assistant training program and identify areas of potential enhancement to improve the management of obstetric emergencies.

This knowledge may help to inform the future design of the EmOC training program, with an aim to reduce maternal and neonatal mortality and morbidity. It is anticipated that the findings will facilitate the development of sustainable and culturally appropriate, educational interventions that will enhance the education and training of MLHPs at local, national, and international levels. This work intends to contribute to addressing Sustainable Development Goal 3, Target 1 of reducing maternal mortality to 70:100,000 live births in low‐income countries (United Nations Development Programme, 2018).

## 2 BACKGROUND

The WHO (2019a) states that over 800 women die each day from preventable causes related to pregnancy and childbirth. Many of these deaths occur in low and lower middle‐income countries, predominately residing in Sub‐Saharan Africa and Southern Asia. Located in the western portion of mainland Southeast Asia (SEA), Myanmar is a lower middle‐income country with an estimated population of 54 million (World Bank, 2021). For many of its independent years, Myanmar has experienced considerable ethnic tensions resulting in one of the world's longest running ongoing civil wars (Bertrand, 2022). A recent intensification of political unrest has resulted in a rapid increase in the number of displaced people and increased levels of poverty. This in conjunction with the effects of the COVID‐19 pandemic has resulted in Myanmar now being considered one of the most fragile settings globally in 2022 (Haken et al., 2022).

The fragility of any given context is continually changing and fluctuates in response to natural disasters, political instability, and poverty levels. People living in fragile settings often face overlapping and interconnected challenges (Relief International, 2023). Families living within remote regions of Myanmar are faced with limited access to quality healthcare and education, poverty, political instability, limited internet access and barriers to economic development, such as a lack of roads and public transport. Even before the recent intensification of political unrest and the COVID‐19 pandemic, the maternal mortality rate (MMR) was 250:100,000 live births (WHO, 2019b). Recent, ongoing disruptions to both health services and health education, means this figure is now likely to be significantly higher.

Provision and uptake of maternity care in Myanmar have been associated with geographical location, level of education, household income and access to transport (Milkowska‐Shibata et al., 2020). Myanmar's Ministry of Health and Sports (MoHS) has increased the number of health worker posts (Saw et al., 2019); however, hard‐to‐reach areas controlled by ethnic groups often cannot be reached by government services (Davis & Jolliffe, 2016). In hard‐to‐reach areas, it has been reported that one midwife may be providing care for 8–10 villages, with approximately 40–50,000 people (Kernohan et al., 2017). The most recent statistics highlight that the presence of a skilled birth attendant (SBA) during delivery is more common in urban areas (88%), compared to remote areas (52%), with many women in remote areas relying on a traditional birth attendant (TBA) (Ministry of Health and Sports [MoHS] & ICF, 2017).

Major complications which account for approximately 75% of all maternal deaths globally include severe bleeding, infections (usually after childbirth), high blood pressure (pre‐eclampsia and eclampsia), complications from delivery and unsafe abortions (WHO, 2019a). Postpartum haemorrhage (PPH) is the leading direct cause of maternal mortality worldwide and accounts for approximately 30% of all maternal deaths in Myanmar (Than et al., 2017). A key intervention to reduce maternal and neonatal mortality worldwide is timely access to a SBA. In order to provide effective, uninterrupted, and quality care, SBAs should be competent, educated, trained, and regulated to national and international standards (WHO, 2018). The minimum care package required during pregnancy and childbirth to manage potentially life‐threatening complications is referred to as EmOC. The recommended EmOC training is comprised of a shortlist of key interventions and activities that address the main causes of maternal death, stillbirth, and early neonatal deaths (WHO, 2009). Although timely, high‐quality EmOC can reduce maternal and neonatal mortality and morbidity, evidence has highlighted that health workers in low‐ and middle‐income countries (LMICs) may lack the competencies to provide all signal functions (Actis Danna et al., 2020).

A growing body of literature has examined the various educational and training approaches employed to enhance the management of obstetric emergencies. Asian education differs from the more liberal Western culture, often favouring a teacher‐centred, passive approach (Loh & Teo, 2017). Whilst didactic approaches may prove beneficial in the acquisition of knowledge and skills in obstetric emergencies in the SEA context (Cagayan et al., 2022), its effectiveness may be enhanced if used in parallel with more modern approaches, such as problem‐based learning, case‐based learning, and simulation‐based learning (Challa et al., 2021). These modern approaches enable learners to practice their skills in a safe environment and research has highlighted that the application of simulation‐based learning in LMICs may provide a sustainable, feasible and acceptable approach to EmOC education and training (Chou et al., 2022; Meza et al., 2021). Many regions in Myanmar have already incorporated this universally accepted approach of hands‐on experience into their training, with the establishment of skills labs to improve the management of obstetric emergencies (LoLordo, 2016).

In response to the COVID‐19 pandemic, medical education was forced to rapidly transition online and an acceleration in the utilization of technology was required globally. Many high‐income countries (HICs) successfully transitioned with the aid of novel approaches, such as mixed‐reality and videoconferencing (Stojan et al., 2022). Whilst stable settings allow for this integration of technology, the COVID‐19 pandemic may have potentially increased the barriers to quality education and training in LMICs (Connolly & Abdalla, 2022). It is not fully known how the COVID‐19 pandemic has affected EmOC education and training in remote, fragile, and politically unstable contexts; therefore, further research is warranted with an aim to narrow the gap of inequality.

In an aim to ensure health workers are equipped with the knowledge and skills required to deliver high quality, evidence‐based care, culturally relevant and effective EmOC education and training is fundamental. Further research will provide a deeper understanding of the unique educational and training needs within remote, fragile communities. This will aid in the development of culturally appropriate, sustainable approaches to enhance the management of obstetric emergencies. In view of this, the following research question is proposed: Are there areas within a medical assistant training program, located in a remote, fragile region of Southeast Asia, that could be enhanced to improve the management of obstetric emergencies?

## 3 PROTOCOL

### 3.1 Research aims and objectives

The aim of this research is to evaluate the current level of EmOC education and training in a medical assistant training program and identify if there are areas of potential enhancement to improve the management of obstetric emergencies in remote, fragile settings. It will explore the contextual and cultural factors impacting on the acceptability and feasibility of various educational and training approaches in that setting.

The following objectives are proposed:

- To critically review research on EmOC education and training in SEA. This will include methods, modes, adaptability, and theoretical constructs to identify culturally acceptable and adaptable methods or approaches for use in fragile settings.
- To review the current level and content of EmOC education and training in the medical assistant curriculum compared to relevant professional standards.
- To explore the student experience and perception of EmOC education and training.
- To assess the preparedness of the education and training in meeting the practice needs of medical assistants in their post graduate role.
- To identify areas of best practice within the EmOC education and training.

## 4 METHODS AND ANALYSIS

### 4.1 Research approach and theoretical framework

Operating from an interpretive perspective, an inductive approach has been selected. Therefore, this ethnographic study is exploratory and will adopt a multi‐methods approach. Following a review of potential candidate theories, this study will be framed on the Knowledge, Skills, and Attitude Framework (Anderson & Krathwohl, 2001) and underpinned by experiential learning theory (ELT) which states “learning is the process whereby knowledge is created through transformation of experience” (Kolb, 1984, p. 38).

An in‐depth analysis will aim to identify the extent to which the four models of learning defined by ELT (abstract conceptualization, concrete experience, active experimentation, and reflection observation) are incorporated into the medical assistant training curriculum. It will explore individual learning styles to gain an understanding of how students best acquire information and if effective retention of knowledge and skills is achieved.

The rationale for using ELT was based on the known cultural traditions for teaching and learning within this population. The parameters of ELT in this context will permit a deeper understanding of the interplay between information acquisition (knowledge), practice (skills in action) and culture (learned modes of behaviour). In addition, this study will also explore the unique overlapping and interconnected developmental challenges facing Myanmar at present and how these impact the implementation of ELT in this context.

Originally criticized for its exclusion of impacting social and cultural factors, Kolb's later research addresses these impacting factors through external validity evidence (Kolb & Kolb, 2013). The application of ELT provides a useful approach to evaluate EmOC education and training in this context as it provides a blend of traditional and hands‐on learning. ELT may offer opportunities for developing skills in continuing professional development (CPD), which is often neglected in LMIC settings (Chamane et al., 2019). Therefore, this study will also evaluate the acceptability of ELT in terms of the program's contribution to life‐long learning.

### 4.2 Study design overview

This ethnographic study is comprised of three stages, adopting a multi‐methods approach. Stage one aims to evaluate the current level of EmOC education being delivered as part of the medical assistant training program. This will be assessed through documentary analysis and interviews (online or face‐to‐face) with educators and trainers. Stage two will explore student experiences using in person focus groups, facilitated by an external trainer involved in delivering the medical assistant training program. Stage three will use a reflective field diary to provide insight into the lived experience of postgraduate students. Data from stages one to three will be analysed using thematic analysis (Braun & Clarke, 2021) and the full data set will be triangulated and analysed using the READ approach; (1) ready your material, (2) extract data, (3) analyse data and (4) distil your findings (Dalglish et al., 2020).

### 4.3 Data collection

**FIGURE 1:**
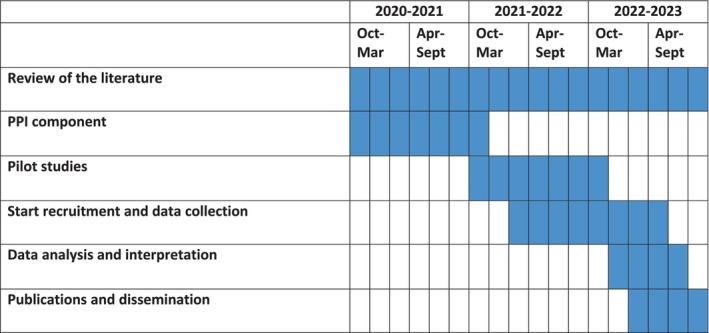
GANTT chart illustrating the study timeline (an ethnographic study evaluating emergency obstetric care education and training in a remote, fragile region of Southeast Asia).

Data collection will be conducted between May 2022 and May 2023. Figure 1 illustrates the study timeline.

### 4.4 Review of the literature

Research on EmOC education and training in SEA will be critically reviewed. This will include methods, modes, adaptability, and theoretical constructs to identify culturally acceptable and adaptable methods/ approaches for use in the Myanmar setting.

A systematic review, building on Ameh et al. (2019) will be conducted. They evaluated the effectiveness of EmOC education and training on a global scale between 1997 and 2017 (inclusive), concluding that significant improvements in healthcare provider competence can result from short competency‐based training in EmOC. They highlighted a paucity of research within the SEA context. Therefore, a systematic review will be conducted from 2018 onwards and will use the Preferred reporting items for systematic reviews and meta‐analysis (PRISMA) (Page et al., 2021). A combination of free‐text and Medical Subject Headings (MeSH) terms will be used in the search strategy including terms in four categories relating to (1) types of health personnel, (2) types of obstetric emergencies, (3) delivery methods of education and training, and (4) countries of SEA.

To guide this work, the Knowledge, Skills, and Attitude framework will be applied (Anderson & Krathwohl, 2001). The eligibility criteria will be developed using the Cochrane PICOS framework (Higgins et al., 2022) (Table 1). Studies will be eligible if they address a change in knowledge and/or skills and/or if they assess participants' attitude (feelings, emotions, beliefs, and values) towards their education and/or training. The following databases will be systematically searched: CINAHL, MEDLINE, ProQuest Dissertations & Theses and Scopus. The grey literature and websites of key international organizations known to be involved in EmOC education and training in SEA will also be searched for relevant publications. Reference lists of all eligible studies and chain tracking will be used to identify studies not already included. To identify any new relevant publications, search alerts will be set up.

**TABLE 1:** Eligibility criteria using the PICOS framework.

PICOS	Description
Population	All categories of primary HCWs and/or students working and/or studying in SEA (Brunei, Myanmar [Burma], Cambodia, Timor‐Leste, Indonesia, Laos, Malaysia, Philippines, Singapore, Thailand, and Vietnam)
Interventions	EmOC education and training in SEA (tertiary education environment, clinical setting, or combination of both). There will be no limitation applied to program duration or intensity
Comparisons	This review will consider studies that compare the intervention to alternative or different interventions or the absence of interventions
Outcomes	Change in knowledge and/or skill and/or participants attitude (feelings, emotions, beliefs, and values) towards their education and/or training
Study design	Experimental and quasi‐experimental, including RCTs, Non RCTs, before and after studies, prospective and retrospective cohort studies, case control studies, analytical cross‐sectional studies, and feasibility studies

*Note*: An ethnographic study evaluating emergency obstetric care education and training in a remote, fragile region of Southeast Asia—review of the literature.

### 4.5 Stakeholder involvement

Stakeholders involved in delivering the EmOC education and training have been consulted in a series of zoom meetings with the researcher team. These meetings generated discussion on the proposed research, while ensuring its relevance within the Myanmar context.

### 4.6 Study setting

Participants will be recruited through an organization delivering a medical assistant training program in a remote, fragile region of Myanmar. The vision of this organization is to provide people living within remote regions with access to high quality healthcare. Students undertake training in small clinics located in remote and mountainous regions of Myanmar, covering a range of services, including adult medicine, paediatrics, and obstetric care. Medical assistants in this setting undertake tasks usually carried out by doctors, nurses, and midwives. The overall medical assistant training program is now recognized and accredited by the regional health department.

### 4.7 Participant sample

The sample of this study will be drawn from local stakeholders, EmOC educators/trainers, doctors, midwives, any other individuals involved in the delivery of the curriculum, student medical assistants and post graduate medical assistants. Participants will be recruited via a named gatekeeper affiliated with the organization delivering the medical assistant training program. EmOC educators/trainers and student medical assistants will be recruited via purposive sampling. Additional criteria such as ‘year of training’ will be applied when conducting focus groups with student medical assistants to ensure participants have a similar foundation. Graduate medical assistants working in the field will be recruited through convenience sampling due to their widespread geographical location in addition to determining their level of EmOC exposure, post‐graduation. The inclusion and exclusion criteria for this study is outlined in Table 2.

**TABLE 2:** Inclusion and exclusion criteria (An ethnographic study evaluating emergency obstetric care education and training in a remote, fragile region of Southeast Asia).

	Inclusion criteria		Exclusion criteria
Stage 1	Educators/trainers involved in the delivery of EmOC education and training	All participants age > 18 years old	Any student or graduate medical assistant who is not currently involved in the program
Stage 2	Students enrolled in years 4 and 5 (EmOC education and training is incorporated into the medical assistant training program in years 4 and 5)		
Stage 3	Graduated medical assistants (who have completed the medical assistant training program being evaluated) with a minimum 6 months working in the field		

## 5 DETAILED STUDY PLAN

**FIGURE 2:**
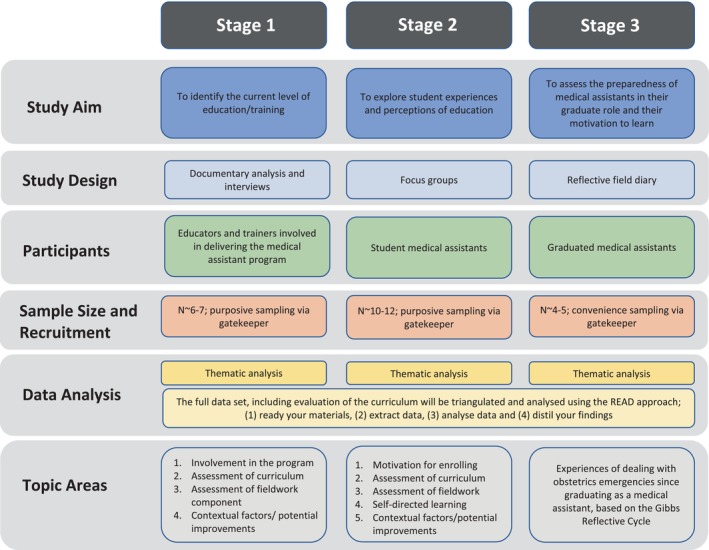
Overview of study design (An ethnographic study evaluating emergency obstetric care education and training in a remote, fragile region of Southeast Asia).

This study is comprised of 3 stages, adopting a multi‐methods approach. A combination of documentary analysis, interviews, focus groups and a field diary will be used to answer the research question. The study design is illustrated in Figure 2.

### 5.1 Stage 1: Background data

Stage one will aim to assess the level and content of EmOC education and training in the medical assistant training program. This will be achieved through documentary analysis of the current curriculum and discussion with the team responsible for the delivery of the program. An English version of the curriculum will be obtained in addition to discussion with key stakeholders to identify which framework(s) it is based on. The current curriculum will then be cross‐examined to identify any inconsistencies or similarities with key global documents relating to intrapartum birth. This stage will also involve discussions with educators/trainers involved in EmOC education and training on the medical assistant training program to gain a thorough understanding of the current curriculum and assessment process. 6–7 participants will be recruited for this component of the study. Interviews will be conducted online or face‐to‐face. Interviews conducted online will be via a secure platform and all interviews will be audio recorded with permission.

### 5.2 Stage 2: Student experience

Stage two will explore the student experience and perception of their EmOC education and training on the medical assistant training program. Student medical assistants will be invited to participate in an in‐person focus group, facilitated by an external trainer involved in delivering the program. Initially two focus groups will be conducted (focus group 1: students enrolled in year 4; focus group 2: students enrolled in year 5). Ideally, each focus group will contain 5–6 students. Focus groups will be conducted until data saturation has been achieved. Students will be audio recorded with permission, using a Dictaphone, in English. A translator will be available if required. The audio recording will be returned to the lead researcher via a secure platform.

### 5.3 Stage 3: Learning fit for purpose

Stage three will assess the preparedness of the education and training in meeting the practice needs of medical assistants in their post‐graduation role. It will explore the lived experience of medical assistants following six months working in the field. It is proposed to include 4–5 graduated medical assistants. To capture these case studies, participants will be asked to keep a diary for three months reflecting on their experiences of dealing with obstetric emergencies. They will be asked to complete a diary entry every time they experience an obstetric emergency over this 3‐month period. This stage will also assess whether the current EmOC curriculum has motivated them to partake in lifelong learning. This will be achieved by exploring what types of additional learning (including self‐directed reading and attendance at training days) the medical assistant has undertaken since graduating. The field diary will be based on the Gibbs Reflective Cycle (Gibbs, 1988) and will allow graduates to learn and plan from what they felt went well or could have been improved in the management of the obstetric emergency.

### 5.4 Recruitment and sample size

Participants will be identified via the named gatekeeper, who is the Midwife Program Director on the medical assistant training program. The gatekeeper will approach participants (letter or email, depending on the resources available in their setting) to invite them to take part in the study. A copy of the participant information sheet (PIS) will be attached to the letter/email of invitation, providing potential participants with further details of the study. If they wish to proceed, they are advised to contact the PhD researcher to formally gain consent. Due to the small cohorts within this study, it is not expected that over‐recruitment will be an issue. In addition, by utilizing interviews and focus groups, it is anticipated that attrition should also not be an issue.

### 5.5 Data analysis

Data obtained from stage one and two will be transcribed verbatim by the lead researcher. Transcripts will be sent to participants for verification and participants will be offered the opportunity to edit their responses and provide additional information if appropriate. Data from stages one to three will be analysed using thematic analysis (Braun & Clarke, 2021). The full data set, including evaluation of the curriculum, will be triangulated, and analysed using the READ approach (Dalglish et al., 2020); (1) ready your materials, (2) extract data, (3) analyse data and (4) distil your findings. NVIVO software will be utilized in the analysis of data.

### 5.6 Recommendations and dissemination

The research team will work in partnership with key stakeholders to identify areas of potential enhancement and propose strategies to incorporate into their current EmOC education and training programme. This aims to not only increase competence and confidence among clinicians but has the potential to propose a framework of education that may be disseminated to other healthcare providers such as SBAs and TBAs in future projects. The involvement of key stakeholders aims to ensure these strategies are culturally tailored and increase sustainability on completion of this study.

### 5.7 Ethical considerations

Institutional ethical approval was obtained from a university in October 2021 and inter country regional access was gained following adherence to their local ethical requirements. This was facilitated by the Southeast Asia research lead from the wider ESRC BAB project (Dr. Lesley Dornan).

### 5.8 Participant burden

The study is designed to ensure minimal disruption to the daily lives of participants. This was confirmed during the pilot tests. Each participant is undertaking one stage of the data collection process, reducing the time demands placed upon them and minimizing any interference with their daily work. Interviews and focus groups will last approximately 45 minutes and will be arranged at a time convenient to the participant. On average, graduated medical assistants took 30 min to complete each diary entry in the pilot test. In response to the busy demand's participants are placed under in their daily duties, a flexible approach will be taken to reduce any additional burden.

### 5.9 Consent

All participants will be required to complete a consent form after reading details about the project and having adequate time to ask questions. Written and verbal consent will be required from all participants prior to engagement in the study. Consent will be taken in English and or any recognized regional language, with assistance from the named gatekeeper. Participants will be informed of their right to withdraw from the study at any time.

### 5.10 Sensitive data

Most of the data will not be of a sensitive nature; however, participants may identify areas of poor practice. The legal and medical delivery of medical care in Myanmar is very different to Western Countries (for example, disclosure of poor practice in the UK is reportable to the Nursing and Midwifery Council); however, guidelines for good practice are in place by participating clinics and will be followed at all stages of data collection. Any incidents that arise will be reported to the participating clinic coordinator, leadership team and in keeping with the rules of the clinic and local health department. If participants disclose poor practice that does not require reporting to the above channels, this will be addressed as a learning event to reduce the chance of it happening again. Graduated medical assistants meet regularly with the teaching team, providing a learning opportunity in which any concerns can be discussed. Additionally, graduated medical assistants have access to the internet and phones, enabling them to consult with the teaching teams located within participating clinics if they encounter any difficult clinical cases.

### 5.11 Confidentiality

In view of the current political instability in Myanmar, data security is an important aspect of this study. Data collection may include both written (hard copy in diary) and electronic data. In stage 3, the field diary is a resource for the student, and therefore they can keep their diary at the end of the study and do with it as they wish. Participants will be prompted to send a copy of their diary entries monthly. This can be done by scanning the diary entry or taking a picture and sending it via a secure network depending on the availability within their setting. Electronic data will be secured on password protected and encrypted computers and backed up on a university server with restricted access (research team). All data will be kept in accordance with the 2018 General Data Protection Regulation (GDPR). The audio file will be destroyed once transcribed and transcripts will be kept in a password‐protected university electronic file in the anonymized format. All research data will be kept for a minimum of 10 years after the end of this study as per university guidelines.

Limits to confidentiality would involve disclosure of criminality or breaches of professional codes of conduct, for example a risk of harm to yourself or others, or professional malpractice. In such cases incidents will be reported to the participating clinic coordinator, leadership team and in keeping with the rules of the clinic and local Health Department. The consideration of ethical issues will be paramount at all stages of the study to ensure the likely benefits of the research outweighs any possible risk to participants.

## 6 RIGOUR

This study will implement the four‐dimension criteria (credibility, dependability, confirmability, and transferability), set out by Lincoln and Guba (1985). To ensure the findings from this study are credible, researchers will build ongoing relationships with key stakeholders involved in delivering the medical assistant training program. This will provide the researchers with a deeper understanding of the context being studied, ensuring the research is culturally relevant and the methods of data collection are feasible. A multi‐methods approach will also be utilized, and the full data set will be triangulated. Investigator triangulation will be adopted, with at least two members of the research team making decisions on coding, analysis, and interpretation. In an aim to strengthen the creditability of the findings, member checking will also be adopted, in that transcripts from interviews and focus groups will be returned to participants for comments and/or corrections. This study will involve feeding back data to key stakeholders and working in close partnership to identify areas of potential enhancement. This process of member checking aims to strengthen the data. The transferability of the research will be facilitated through a thick description of the participants context and the research process, enabling the reader to make a transferability judgement. The dependability and confirmability of the study will be ensured through an audit trial, in which there will be transparency at all stages, from data collection to the reporting of findings.

Reflexivity will also be considered, with the lead researcher keeping a diary to examine their own conceptual lens, any assumptions, preconceptions, and values. This will enable the lead researcher to consider if these have impacted on any research decisions or interpretations. The research is led by an experienced team, representing both genders and with varying levels of research and clinical experience. One member of the research team has lived in Southeast Asia for an extended period and has contributed to our understanding of the unique cultural and contextual factors in the region.

## 7 DISCUSSION

There are many practical challenges associated with conducting research in remote, fragile settings and these have been pivotal in the design of this study. In view of the current political situation in Myanmar and the COVID‐19 pandemic, the decision was made to conduct data collection online, except for one face‐to‐face interview, which will be conducted in line with current government regulations and guidelines on social interaction and distancing. In response to the continually evolving political situation and restricted internet access, an ethical amendment was approved to conduct in‐person focus groups. These will be facilitated by an external trainer involved in the delivery of the medical assistant training program. Various safety measures will be put in place to mitigate the potential risk of harm to researchers. Participant safety and sensitivity to the local situation will be paramount at all times and a flexible approach to data collection will be taken. If necessary, all data collection will be temporarily postponed.

This study is part of a wider project ‘Birth Across the Borders’, and local approval was facilitated by the Southeast Asia research lead from ESRC BAB project (Dr Lesley Dornan). This has resulted in assuring the research team that recruitment of participants for this study will not be an issue. A series of zoom meetings with key stakeholders within the organization, who are familiar with the culture ensures that the data collection methods are feasible and that the study is culturally relevant. In year one of the medical assistant training program, students are given English language classes; therefore, it is anticipated that participants will have a good grasp of the English language, however, as a back‐up a translator will be available on request. The translator will have a range of dialects from the region and will be working under the confidentiality agreement, as guided by the approving university ethics committee. Extra time will be allocated for interviews to allow for any loss of connection or internet issues. There will be no financial payment for taking part in the study; however, any cost incurred will be reimbursed, such as travel costs, recording equipment and printing.

## 8 LIMITATIONS

The medical assistant training program we will be evaluating is currently being delivered by an organization working in a remote, fragile region of Southeast Asia. Key stakeholders within the organization have acknowledged that they may have access to resources and infrastructure unique to that context. However, the focus of this study is to identify effective educational and training approach to deliver EmOC education and training. The study will focus on cultural and contextual factors that may impact on the delivery of EmOC training program, many of which will be similar in other remote, fragile regions at local, national, and international levels.

The process of ethnography commonly involves the direct observation of people in their natural environment, often for an extended period (Reeves et al., 2013). However, in view of the COVID‐19 pandemic and political instability within the region being studied, this is not feasible. Therefore, stage 2 will be facilitated by an external trainer involved in delivering the EmOC component of the medical assistant training program. The research team in collaboration with the gatekeeper chose an external visitor from the UK, who is not part of the main teaching team and is only visiting for a short period, to help minimize the risk of coercion and openness.

## 9 CONTRIBUTION TO LITERATURE

In response to a paucity of research, this study aims to evaluate EmOC education and training in a remote, fragile region of Myanmar. We anticipate that many best‐in‐class training programs and approaches adopted by high‐income countries (HICs) and other low‐ and middle‐income countries (LMICs) may not be transferable to the current context in Myanmar. This is due to the complex development challenges facing Myanmar at present, such as a lack of resources and infrastructure, political instability, lack of quality education and barriers to economic development. However, existing literature suggests that a hybrid approach to EmOC education and training may be beneficial, incorporating traditional didactic approaches with more modern approaches such as simulation‐based learning. This hybrid approach to EmOC education and training has been shown to be beneficial in both HICs and LMICs. The introduction of Practical Obstetric Multi‐Professional Training (PROMPT, 2023) has had a significant impact on reducing maternal and neonatal mortality and morbidity worldwide. There was a 34% reduction in maternal deaths following the implementation of PROMPT in Zimbabwe, and its benefit in the SEA context has been demonstrated with a 26% reduction in maternal deaths in the Philippines.

We foresee an enhanced utilization of technology within the medical assistant training program in response to the COVID‐19 pandemic. However, in view of a lack of resources and infrastructure, we anticipate that challenges may have arisen during this transition. This study aims to add to existing literature, focusing on gaining a deeper understanding of the unique contextual and cultural factors impacting on EmOC education and training in this setting. Of significance, education, and training in EmOC is a dynamic process, requiring regular refresher training and periodic refinement. This is of particular importance in fragile settings, were training programs may benefit from a flexible curriculum.

## 10 CONCLUSION

It is proposed that this study will contribute new knowledge and understanding regarding EmOC education and training in a remote, fragile region of Southeast Asia. It has the potential to facilitate the development of sustainable, culturally appropriate, educational interventions, which may enhance the management of obstetric emergencies. This study is an essential component within the wider ESCR funded, BAB project. The aim is to support SEA in achieving Sustainable Developmental Goal 3 (Target 1) in reducing maternal mortality to 70:100,000 live births in low‐income countries (United Nations Development Programme, 2018).

## AUTHOR CONTRIBUTIONS

Rachel Campbell, George Kernohan, Lesley Dornan, Marlene Sinclair: Conceptualization. George Kernohan, Lesley Dornan, Marlene Sinclair: Funding acquisition. Rachel Campbell, George Kernohan, Lesley Dornan, Marlene Sinclair: Investigation. Rachel Campbell, George Kernohan, Lesley Dornan, Marlene Sinclair: Methodology. Rachel Campbell, George Kernohan, Lesley Dornan, Marlene Sinclair: Project administration. Rachel Campbell, George Kernohan, Lesley Dornan, Marlene Sinclair: Resources. George Kernohan, Lesley Dornan, Marlene Sinclair: Supervision. Rachel Campbell: Writing‐original draft preparation. George Kernohan, Lesley Dornan, Marlene Sinclair: Writing‐review and editing.

## FUNDING INFORMATION

This research was funded by the Northern Ireland Department for the Economy (DfE) Postgraduate Studentship (2020/2021–2022/2023).

## CONFLICT OF INTEREST STATEMENT

The authors declare no conflicts of interest.

## Data Availability

Data sharing is not applicable to this article as no new data were created or analyzed in this study.
